# Haplotype Networking of GWAS Hits for Citrulline Variation Associated with the Domestication of Watermelon

**DOI:** 10.3390/ijms20215392

**Published:** 2019-10-29

**Authors:** Vijay Joshi, Suhas Shinde, Padma Nimmakayala, Venkata Lakshmi Abburi, Suresh Babu Alaparthi, Carlos Lopez-Ortiz, Amnon Levi, Girish Panicker, Umesh K. Reddy

**Affiliations:** 1 Department of Horticultural Sciences, Texas A&M University, and Texas Texas A&M AgriLife Research and Extension Center, Uvalde, TX 78801, USA; Vijay.Joshi@ag.tamu.edu; 2 Department of Biology and Gus R. Douglass Institute, West Virginia State University, Institute, WV 25112, USA; suhas.shinde@wvstateu.edu (S.S.); vabburi@wvstateu.edu (V.L.A.); salaparthi@wvstateu.edu (S.B.A.); carlos.ortiz@wvstateu.edu (C.L.-O.); 3 U.S. Department of Agriculture-Agricultural Research Service, U.S. Vegetable Laboratory, Charleston, SC 29414, USA; Amnon.Levi@ars.usda.gov; 4 Center for Conservation Research, Alcorn State University, 1000 ASU Drive, Lorman, MS 39096, USA; panicker@alcorn.edu

**Keywords:** citrulline, genome-wide association study, haplotype, watermelon, *acetolactate synthase*, *ferrochelatase*

## Abstract

Watermelon is a good source of citrulline, a non-protein amino acid. Citrulline has several therapeutic and clinical implications as it produces nitric oxide via arginine. In plants, citrulline plays a pivotal role in nitrogen transport and osmoprotection. The purpose of this study was to identify single nucleotide polymorphism (SNP) markers associated with citrulline metabolism using a genome-wide association study (GWAS) and understand the role of citrulline in watermelon domestication. A watermelon collection consisting of 187 wild, landraces, and cultivated accessions was used to estimate citrulline content. An association analysis involved a total of 12,125 SNPs with a minor allele frequency (MAF)>0.05 in understanding the population structure and phylogeny in light of citrulline accumulation. Wild egusi types and landraces contained low to medium citrulline content, whereas cultivars had higher content, which suggests that obtaining higher content of citrulline is a domesticated trait. GWAS analysis identified candidate genes (*ferrochelatase* and *acetolactate synthase*) showing a significant association of SNPs with citrulline content. Haplotype networking indicated positive selection from wild to domesticated watermelon. To our knowledge, this is the first study showing genetic regulation of citrulline variation in plants by using a GWAS strategy. These results provide new insights into the citrulline metabolism in plants and the possibility of incorporating high citrulline as a trait in watermelon breeding programs.

## 1. Introduction

Non-protein amino acids present in legumes, fruits, seeds, and nuts are ubiquitous in the human diet. Besides containing several health-promoting bioactive compounds, fruits accumulate substantial amounts of free non-protein amino acids. With a diversity of phytochemicals such as carotenoids, flavonoids, and triterpenoids, watermelon (*Citrullus lanatus* var. *vulgaris*) fruits also accumulate a substantial amount of a non-protein amino acid—citrulline. Scientific studies have demonstrated several health benefits of citrulline, such as anti-atherosclerotic (hardening of the arteries) effects, reduction of aortic blood pressure and stiffness in individuals with hypertension and cardiovascular diseases, improving lipid profiles by lowering cholesterol, lowering inflammation, and increasing athletic performance [1,2,3,4,5]. Additionally, many recent reviews have covered the clinical impacts of citrulline administration on human health in detail [6,7,8]. Consumption of watermelon has been shown to increase plasma arginine concentrations in adults [9,10]. Watermelons are readily available in most parts of the world, including dry and hot areas of the African continent, where most fruits would not thrive. As watermelon is a nutrient-dense fruit, it is recommended as part of a healthy meal plan as per the U.S. Department of Agriculture’s MyPlate guidelines. In plants, citrulline has been suggested to have a functional role in the nitrogen transport and maintenance of cellular osmolarity during abiotic stresses in plants [11,12]. Members of the Cucurbitaceae family are generally considered to contain relatively large amounts of free citrulline, although watermelon accumulates the highest quantities [13]. Citrulline content in watermelons is spatially and developmentally regulated, with the highest values occurring at fruit maturity [14,15,16,17,18]. Unlike its investigation in plants, citrulline regulation has been extensively studied in the mammalian, prokaryotic, and yeast systems [19]. In the absence of functional nitric oxide synthase (NOS), citrulline in plants is synthesized as a metabolic intermediate during arginine biosynthesis by using carbamoyl phosphate and ornithine. Several studies reported the presence of genotypic variation for citrulline content in a selected set of cultivated watermelon varieties [11,16,18,20,21]. A moderate to high range of heritability [21,22] for citrulline content within cultivated watermelons implies its possible genetic improvement with selective breeding.

A complex compartmentalized network of genes coordinates several metabolic pathways to regulate amino acid metabolism in plants. To understand the molecular regulation and genetic inheritance of amino acids, these networks can be unraveled with the availability of whole-genome sequences and other functional resources. Over the last 2 decades, genome-wide association studies (GWASs) have continued to be the favorite tool to identify causal genetic loci of quantitative or qualitative traits in diverse germplasm collections exploiting evolutionarily conserved recombination events. Several GWAS studies have successfully identified candidate genes by using primary metabolites such as amino acid profiles [23,24,25]. As a naturally rich source of citrulline and with the advent of a newly available genome [26], watermelon could serve as an excellent model to study the evolutionary, biochemical, and molecular determinants of citrulline metabolism and regulation. We previously analyzed genome-wide diversity in watermelon by using a large set of SNPs from accessions collected around the world [27,28] to identify genetic loci controlling traits such as fruit firmness, trichome density and length, fruit length, width, rind thickness and soluble solids [29].

However, we lack information on the genetic basis for variability in citrulline content in watermelon fruits. Our studies with GWAS in watermelon will allow us to estimate population structure and linkage disequilibrium (LD), connecting the variation in the genome with the citrulline content in watermelon germplasm. To understand the evolutionary significance of citrulline in watermelon domestication and its possible genetic regulation, this current study involved (1) characterizing genotypic and phenotypic diversity for citrulline content in a watermelon diversity panel representing wild types, landraces and cultivars; (2) identifying candidate genes significantly associated with citrulline variation; and (3) validating the role of selected genes showing significant association by quantitative real-time-PCR analysis.

## 2. Results

### 2.1. Phenotypic and Geographic Variation in Citrulline Content

**Figure 1:**
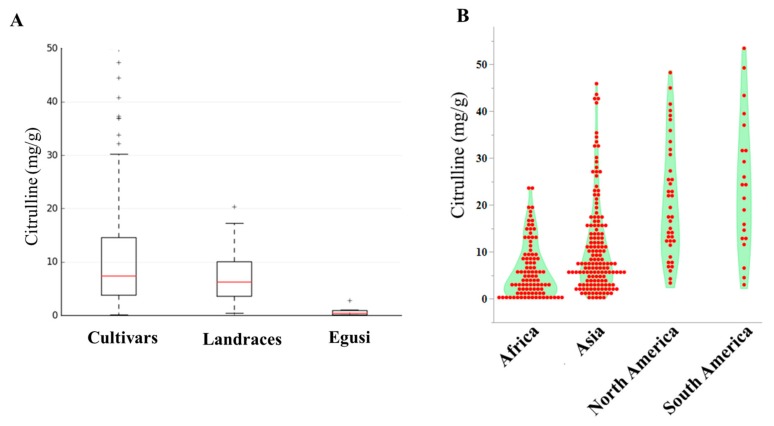
Citrulline content in watermelon accessions. (**A**) Box plots showing the range, mean, and distribution of citrulline content in cultivars, landraces, and egusi types. (**B**) Violin-scaled contour map showing world geographic variation in citrulline content across the accessions.

The current study examined the collection of 144 *Citrullus. lanatus* var. *vulgaris* (sweet watermelons) accessions, 34 semi-wild types (hereafter called landraces), and 9 accessions belonging to *Citrullus. mucasospermus* (egusi) (Table S1). Our collection contained plant introduction (PI) accessions from Africa, Asia, Europe, North America, and South America. African types were from Algeria, Botswana, Egypt, Ethiopia, Ghana, Kenya, Liberia, Mali, Nigeria, Senegal, South Africa, Sudan, Zaire, Zambia, and Zimbabwe. The detailed distribution and quantitative variation of citrulline content of accessions used in this study are in Figure S1 and Table S1. The mean, range, and distribution of citrulline content in cultivars, landraces, and egusi types are shown in Figure 1A. The means of citrulline content were 11.08, 7.35, and 0.8 mg/g in cultivars, landraces, and egusi types respectively, which indicates high citrulline content as a feature of cultivars. The average citrulline content in the accessions from North and South America was significantly higher (*p* ≤ 0.0001) than the accessions from Africa (Figure 1B). Among the selected accessions, mean citrulline content was 10.0 ± 0.04 mg/g (ranging from 0.10 to 47.3 mg/g). The dispersion of free citrulline across the accessions was leptokurtic (kurtosis = 3.27) and asymmetrical (skewness = 1.8). Accessions PI 559993, PI 426625, PI 560020, and PI 526238 had the lowest free citrulline in flesh and Garrisonian, Cole’s Early, and PI 442826 had the highest. Restricted maximum likelihood/best linear unbiased prediction (REML/BLUP) [30,31], variance component estimation revealed significant variation in citrulline content within accessions. The estimates of heritability based on REML analysis were high (83%) for citrulline content, whereas the genetic gain at 5% selection intensity was 9.5%.

### 2.2. Population Structure of Various Accessions Based on Citrulline Content

**Figure 2:**
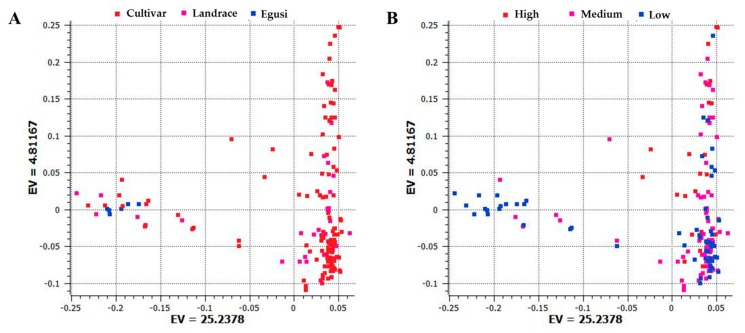
Principal component analysis (PCA) based on the first two components showing the distribution of (**A**) cultivars, landraces, and egusi types; (**B**) and low, medium, and high citrulline content in 187 watermelon accessions by using 1410 single nucleotide polymorphisms (SNPs) generated by genotyping by sequencing. Each dot represents an accession. EV indicates the percentage of explained variance.

To examine divergence across accessions during evolution, analysis of population structure and phylogenetic relationships and PCA were carried out [27]. Using the SNP dataset, we constructed a PCA with the first and second principal components PC1 (25.24) and PC2 (4.81) that separated egusi, landraces, and sweet watermelons (Figure 2A). This PCA also separated low, medium, and high citrulline content types (Figure 2B). Sweet watermelon types contained the highest citrulline content as compared with their ancestral progenitors. Egusi types and landraces contained low to medium citrulline content, whereas cultivars showed increased content, which indicates that high citrulline content is a domesticated trait.

**Figure 3:**
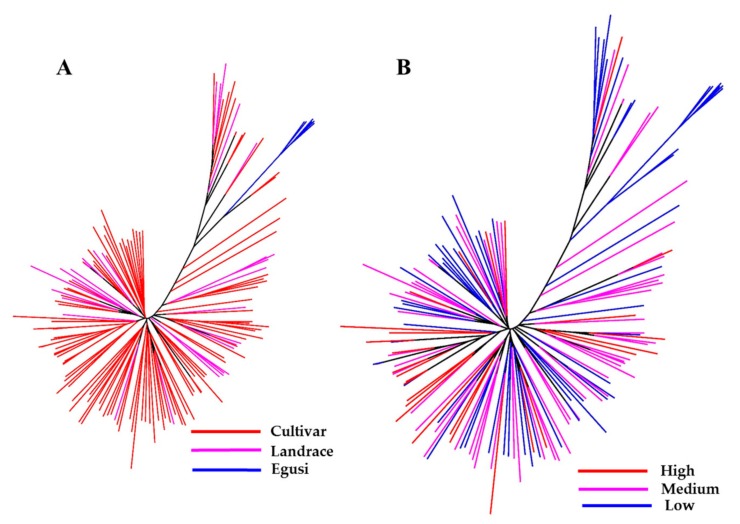
Genetic relationship between a set of 187 watermelon accessions. Neighbor-joining (NJ) tree constructed with 1410 high-quality SNPs explains most of the genetic structure of watermelon germplasm by (**A**) type and (**B**) citrulline content. Accessions in blue-, pink-, and red-colored clades are egusi-types, sweet watermelons, and landraces, respectively. In (**B**), blue, pink, and red clades represent low, medium, and high citrulline content, respectively.

Using the final SNP dataset, we constructed an unrooted neighbor-joining (NJ) tree to infer phylogenetic relationships and understand the distribution of citrulline content across the *C. lanatus* accessions (Figure 3A,B). One group (colored blue in the tree) represents the entire egusi collections. The pink- and red-colored clades represent sweet watermelons and landraces (most from South Africa), many with a hard rind and white flesh resembling an intermediate between egusi and sweet watermelon. This study indicated that most low to medium citrulline-content types are egusi and landraces.

### 2.3. Genome-wide Association Study to Locate Quantitative Trait Loci for Citrulline Content

**Figure 4:**
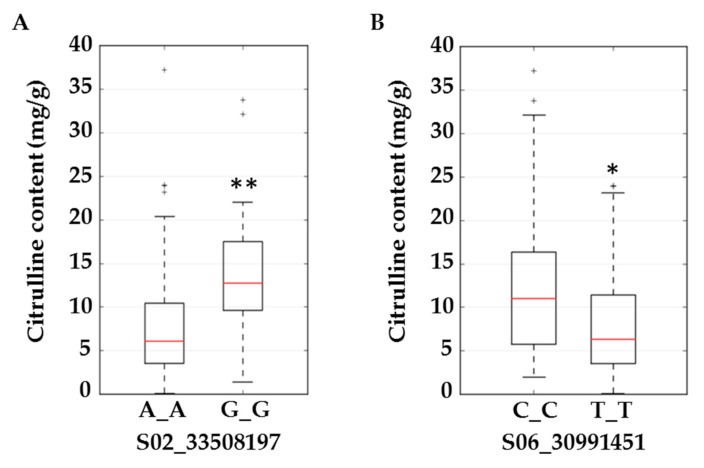
Boxplots for citrulline content (mg/g) in flesh tissue at SNP S02_33508197 located in the intron of *ferrochelatase* (**A**) and S06_30991451 located in an exon of *acetolactate synthase* (**B**). Significant differences (based on the Kruskal–Wallis test) with *p* ≤ 0.01 and *p* ≤ 0.05 are marked with two (**) and one (*) asterisks respectively.

We used a GWAS with 12,125 SNPs to identify alleles that affect citrulline content (Figure 4); individual SNP associations along with the details of major and minor allele frequencies and magnitude of associations are in Table 1 and detailed annotations for all associated SNP markers are in Table S2. We found 12 SNPs associated with citrulline content (Table 1 and Table 2). Significantly associated SNPs for citrulline content were S02_33508197, S02_33508131, S02_28460679, S04_19161720, S04_10803195, S04_19161725, S06_30930976, S06_30991451, S07_12838412, S07_6258382, S09_9172194, and S10_19726131 and were found in ferrochelatase, F-box/LRR-repeat protein 2, Golgi SNAP receptor complex member 2, DNA polymerase I/DNA polymerase I, acetolactate synthase, BAG family molecular chaperone regulator 1, TLC ATP/ADP transporter, protein of unknown function, and phototropic-responsive NPH3 family protein genes, respectively. Biological roles, molecular processes, and the cellular location of these genes are in Table 2.

**Figure 5:**
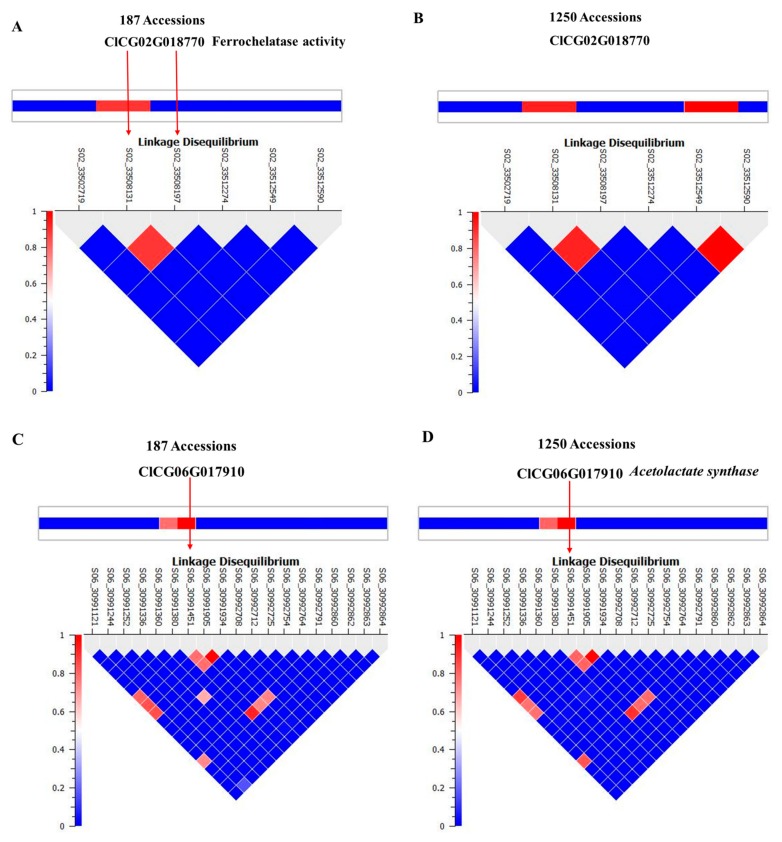
Linkage disequilibrium structure and implicated genomic regions for SNPs aligned with: (**A**,**B**) *ferrochelatase*; (**C**,**D**) *acetolactate synthase*.

We selected *ferrochelatase* (*FC*) and *acetolactate synthase* (*ALS*) genes for further validation. Allelic effects of SNPs located in these genes are in Figure 4. S02_33508197 is located in the intron of *FC*. Allele frequencies for S02_33508197 were 0.83 for AA and 0.17 for the minor allele GG. Average citrulline content was 5 mg/g for the AA-containing genotype and 12.5 mg/g for the GG-containing genotypes (Figure 4A). S06_30991451 is located in an exon of *ALS* and a non-synonymous mutation causing N→S with allele frequencies for TT and CC of 0.79 and 0.21, respectively. Allelic effects for major (TT) and minor (CC) alleles can be noted from the box plot (Figure 4B). Strong LD is noted around the associated SNPs in Figure 5A,C. LD around these two genes was further confirmed from a robust set of 1250 accessions (Figure 5B,D) in a recently published study [26].

**Figure 6:**
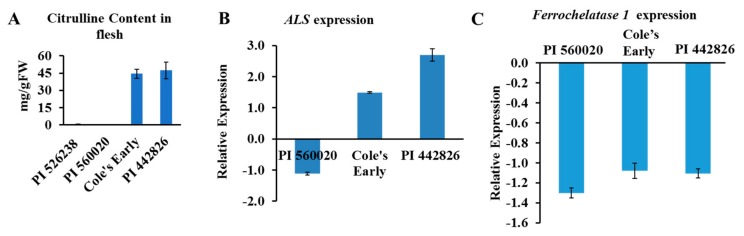
(**A**) Citrulline content and expression of candidate genes in flesh of selected watermelon accessions. Data are means ± SD (*n* = 3). (**B**) Expression of *acetolactate synthase* (*ALS*) and (**C**) *Ferrochelatase 1* genes in flesh. PI 526238 accession, with low citrulline content, was used as a calibrator for relative expression in (**A**). Gene expression was normalized to that of *Actin 8*. Data are means ± SD (*n* = 3).

The watermelon genome has a single copy of *ALS* gene (a large subunit) and two genes that encode *ALS* small subunits (ClCG09G014670 and ClCG03G010140, putative *ALS* small subunits 1 and 2, respectively). The ALS enzyme facilitates the first step in the biosynthesis of branched-chain amino acids (BCAAs; valine, leucine, and isoleucine) in microbes and plants. Moreover, ALS enzyme is inhibited by a group of imidazolinone and sulfonylurea herbicides [32,33,34], thereby preventing biosynthesis of BCAAs. We used real-time quantitative PCR to validate the association of the ALS gene(s) in selected high and low citrulline-content watermelon accessions (Figure 6A). The relative expression of *ALS* mRNA was significantly upregulated in flesh tissues of high citrulline-content watermelon accessions and downregulated in PI560020, with low citrulline content (Figure 6B). In summary, the expression of *ALS* showed strong association with citrulline content in watermelon.

The watermelon genome database search revealed two genes coding for FC enzyme: ClCG02G018770 (appeared in GWAS) and ClCG08G016940. The BLAST search against the *Arabidopsis thaliana* genome revealed that the watermelon ClCG08G016940 and ClCG02G018770 genes are orthologues of AT5G26030 and AT2G30390, labeled *FC1* and *FC2,* respectively.

The mRNA abundance for *FC1* was quantified by qRT-PCR, and we wondered whether *FC1* transcript level was associated with citrulline level in watermelon. Reduced *FC1* transcript abundance was seen in all three accessions (Figure 6C). However, the reason as to why the *FC1* expression was low in accessions with both high and low citrulline content is elusive. We hypothesize that FC1 may act in a spatiotemporal and growth dependent manner. Our data suggest that the *ALS* and *FC* genes may have a strong association with citrulline accumulation. However, future analysis is required to characterize the functions of FC and ALS proteins in citrulline-BCAA metabolism and NO turnover in watermelon.

### 2.4. Haplotyping and Network Analysis of Acetolactate Synthase and Ferrochelatase

**Figure 7:**
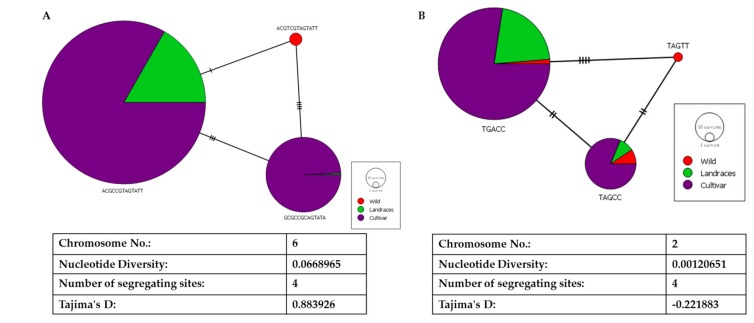
Haplotyping and network analysis of (**A**) *acetolactate synthase* and (**B**) *ferrochelatase* on chromosomes 6 and 2, respectively.

With the four segregating sites in the LD block in *ALS*, we could build a network of haplotypes for egusi (wild), landraces, and cultivated watermelon (Figure 7A). Haplotype ACGTCGTAGTATT had undergone a single nucleotide change to form a landrace haplotype (ACGCCGTAGTATT). GCGCCGCAGTATA has three segregating sites as compared with the landrace haplotype and four segregating sites as compared with the wild haplotype. We noted Tajima’s D as 0.88 and nucleotide diversity of 0.07, indicating a high degree of positive selection around this gene. Contrastingly, for the other causal gene *FC*, we noted reduced nucleotide diversity of 0.01 and negative Tajima’s D (−0.221) indicating purifying selection in evolution (Figure 7B).

## 3. Discussion

The current research aimed to analyze citrulline content evolution by using GBS-generated SNPs across the global collections of sweet watermelon, landraces, and egusi types. Watermelons were cultivated in the northeast of Africa around 5000 years ago [35]. Pictures on Egyptian frescos and seeds found in the grave of King Tutankhamun indicated that the flesh of watermelons was still white and tasted bland [36]. These bland vegetable-kind watermelons were cultivated for thousands of years before sweet watermelons arose [37]. Depictions of cut watermelons throughout the centuries (Giovanni Stanchi 1608 to c. 1675) deviated from the modern watermelons. When explored through flesh of landraces, one can find the evidence for these anthropological records. Because of this, in the current study, landraces are named as semi-wild as the sweet watermelons are quite different. Modern watermelon provides a large amount of water and nutrients, such as sugars, carotenoids, lycopene, minerals, and amino acids, including citrulline. Although citrulline is the most abundant free amino acid in watermelon [12], because of the crop’s commercial value, genetic mapping efforts have mostly focused on sugar content [38,39]. During domestication, dessert watermelons were mostly selected for qualitative traits such as sweetness, flesh color, and rind pattern [40]. Although primitive dessert watermelon landraces, with naturally low sugar content, were expected to be valuable sources of bioactive compounds [41], our study demonstrates that citrulline content is, in general, lower in landraces and egusi types. Modern cultivars from the Americas that are rich in citrulline may have acquired the trait inadvertently during the selection for sweetness or the trait might have an unknown role in adaptation. A positive correlation of sugar content with citrulline was demonstrated in modern watermelon cultivars [18,21,22]. The distinctness of American, European and Asian ecotypes has been described in watermelons [29,42,43], but an independent parallel phenotypic evolution for citrulline during domestication cannot be ruled out [44].

### 3.1. Genetic Characterization of ALS and FC Locus Haplogroups

We genotyped 187 watermelon accessions with a mix of cultivars, landraces and egusi types predominantly collected from Africa, Europe, North America, and Asia with ~12K SNPs. A subset of this data allowed for estimating genomic diversity across the LD around the associated locus among various groups, constructing an NJ tree, resolving population structure, estimating chromosome-wise LD patterns and understanding the extent of population differentiation and haplotype networking in terms of citrulline content in watermelons. This study revealed high nucleotide diversity and Tajima’s D for the *ALS* locus, which showed a strong association with citrulline content. In this study sweet watermelon in South Africa, and most landraces of South Africa clustered with sweet watermelon, which strengthens the argument that the Kalahari Desert could be the center of origin [45,46,47]. Alternatively, egusi types of northeast Africa could be the progenitors of landrace watermelon types because they share segregating sites in the both haplotype networks, thereby indicating a stepwise evolutionary pattern and probably is the second event of domestication. Similar to our observation in this study of haplotype networking in the *ALS* and *FC* loci between egusi and sweet watermelon, Chomicki and Renner [48] reclassified egusi (var. mucasospermus) and sweet watermelons (var. vulgaris) as two different species naming egusi as *C. mucasospermus*. Knowledge of causal genes underlying domestication traits and distribution of functionally diverse alleles in different watermelon populations may be required to better demonstrate the evolutionary route of dessert watermelons [26]. The current SNP-based analysis revealed citrulline accumulation as an important step for the domestication process of sweet watermelons. In this study, we identified 105 private SNPs segregating in sweet watermelons that was not found in related wild species. Such SNPs are valuable because they can be of adaptive importance and would be of immense use for generating passport information for enhancing nutritional traits such as citrulline.

### 3.2. Plausible Role of Candidate Genes Identified in Citrulline Biosynthesis

In eukaryotic cells, FC proteins are located in the mitochondrial inner membrane and facilitate the final step in the heme biosynthetic pathway, inserting a ferrous iron into protoporphyrin IX to yield the heme [49]. In animals, arginine can be a substrate for citrulline biosynthesis via the action of NOS [50,51,52]. However, in plants, arginine-dependent NOS-like proteins are not functionally validated; therefore, the synthesis of citrulline directly from arginine in plants remains debated [18,53,54]. In animals, NOS proteins are soluble hemoproteins that facilitate the conversion of l-arginine to citrulline and NO [55]. NO, a crucial signaling molecule also called a gaseous hormone in plants and animals regulates many physiological and biochemical processes and stress responses in plants (reviewed by Domingos et al. [56]). In animals, NO preferentially binds to hemoproteins in both ferrous and ferric states [57,58], which indicates a strong association of heme synthesis by FC and the arginine–citrulline cycle. We observed that both low and high citrulline content accessions showed reduced *FC1* expression. However, the reason as to why the *FC1* transcript levels were low in accessions with both high and low citrulline content is elusive. We hypothesize that *FC1* expression may be regulated in a spatio-temporal and growth-dependent manner. Further analysis of *FC1* expression in watermelon flesh at various fruit developmental stages may reveal the association of citrulline content and *FC1* transcript abundance.

The ALS complex consists of three pairs of subunits, a large subunit responsible for catalysis, and a small subunit for feedback inhibition. All three genes encoding the putative ALS large subunit (ClCG06G017910), ALS small subunit_1 (ASsu1; ClCG09G014670) and ALS small subunit_2 (ASsu2; ClCG03G010140) proteins were found in the watermelon genome. There are no known direct metabolic links between citrulline and BCAA synthesis (BCAAs) in the canonical or non-canonical systems. Because of the sub-optimal levels of free citrulline in most plants, limited information is available on its relationship with other amino acids. Nonetheless, some studies suggest the possibility of unknown links between citrulline/arginine and BCAA pathways; for example, (1) arginine, a catabolic product of citrulline, is significantly and positively correlated with BCAAs in soybean seeds [59]; (2) levels of BCAAs were increased along with those of citrulline, arginine, and ornithine during conditional downregulation of *target of rapamycin* gene in *Arabidopsis* [60]; and (3) citrulline level was increased in leaves of the rice ALS mutant [61].

## 4. Materials and Methods

### 4.1. Plant Materials, Citrulline Extraction, and Quantification

Selfed seeds of 187 National Plant Germplasm System collections of *C. lanatus* var. *vulgaris* and *C. lanatus* var. *mucasospermus* were grown in the greenhouse. The authors are grateful to R. Jarret, Plant Genetic Resources Conservation Unit, USDA-ARS (Griffin, GA, USA) for providing the seeds of germplasm accessions. Citrulline content in the flesh determined by using three biological replicates for each accession (Table S1). For determining citrulline content, ~20 mg lyophilized flesh tissue for each biological replicate was homogenized by using 3 mm Demag stainless steel balls (Abbott Ball Co., CT, USA) and a Harbil model 5G-HD paint shaker, suspended in 20 mM cold HCl and centrifuged at 14,609× *g* for 20 min at 4 °C. The supernatant was filtered by using 0.45 uM 96-well filters (Pall Life Sciences, NY, USA). The filtrate was derivatized with an AccQ-Fluor reagent kit (Waters Corp., Milford, MA, USA) per the manufacturer’s protocol. UPLC-ESI-MS/MS analysis involved using a Waters Acquity H-class UPLC system coupled to a Waters Xevo TQ mass spectrometer with an electrospray ionization (ESI) probe, Waters ACQUITY UPLC Fluorescence (FLR) detector and Water’s AccQ•Tag Ultra column. The mobile phase consisted of water (0.1% formic acid v/v) (A) and acetonitrile (0.1% formic acid v/v) (B). The column heater was set to 60 °C, and the mobile phase flow rate was maintained at a constant rate of 0.6 mL/min. By using the Waters IntelliStart software, multiple reaction monitoring transitions for citrulline were optimized. The ESI source was operated at 150 °C, with desolvation temperature 450 °C, desolvation gas flow rate 900 L/h and capillary voltage 3.2 kV. Multiple reaction monitoring was performed in the positive mode. Instrument monitoring and data acquisition, integration, and quantification involved using Water’s MassLynx software.

### 4.2. Phylogenetic and Population Genomic Analyses

Phenotypic data were analyzed by using JMP software (JMP Pro 14 (SAS Institute, Cary, NC, SAS http://www.jmp.com/en_us/home.html)). The variance components were estimated by using REML-BLUP analysis. The broad-sense heritability was estimated as H^2^ = δ^2^_g_/( δ^2^_g_ + δ^2^_e/r_), where δ^2^_g_ and δ^2^_e_ are estimated genotypic and error variances, respectively [62]. The genetic gain was calculated as Gs = K × H^2^ × (δ^2^_p_)^−1/2^, where K is the selection intensity at 5% (*k* = 2.056), H^2^ is heritability in a broad sense and (δ^2^_p_)^−1/2^ is the phenotypic standard deviation. Totals of 11,485 SNPs [27] and 16,292 SNPs [26] were combined and further filtered using MAF ≥ 0.01 to identify 12,125 SNPs that have a call rate of 70% in our GWAS panel. For population stratification analysis, we used more stringent cut off for SNP selection based on MAF of 0.1 and 90% call rate. Further informative SNPs were selected by removing SNPs located in a LD block by 50% cut off and also SNPs that deviated from Hardy–Weinberg equilibrium were discarded. A neighbor-joining (NJ) tree for watermelon accessions was constructed by using TASSEL 5.0 [63]. Archaeopteryx [64] was used to visualize and analyze the tree. For analyzing population structure, we used the principal components, or eigenvectors, of principal component analysis (PCA), and corresponding eigenvalues were estimated by using the EIGENSTRAT algorithm [65] with the SNP & Variation Suite (SVS v8.8.1; Golden Helix, Inc., Bozeman, MT, www.goldenhelix.com).

### 4.3. Association Analysis

For GWAS, the population structure Q matrix was replaced by the PC matrix. The PC matrix and identity by descent (IBD) was calculated from LD-pruned SNPs in SVS v8.1.5. GWAS involved a multiple-locus mixed linear model developed by the EMMAX method and implemented in SVS v8.1.5. We used a PC matrix (first two vectors) and the IBD matrix to correct for population stratification. Manhattan plots for associated SNPs were visualized in GenomeBrowse v1.0 (Golden Helix, Inc) (Figure S2). The SNP *p*-values from GWAS underwent false discovery rate (FDR) analysis.

### 4.4. Haplotype Network Analysis

To evaluate the haplotype frequency, we first specified the haplogroups and the frequency with which a determined haplotype appeared in each haplogroup. Then, we determined the genetic constitution as the proportion of SNPs that belong to a haplogroup. The haplotype frequency analysis involved estimating haplotype diversity. The number of haplotypes and the haplotype diversity values were calculated by using DnaSP 5.10 (http://www.ub.edu/dnasp/DnaSP_OS.html) [66]. Haplotype networking and LD calculation were performed by using publicly available SNP data for 1209 *C. lanatus* var. *vulgaris* and 51 *C. mucasospermus* (egusi) [21]. Adjacent SNP pairs within a chromosome underwent LD analysis by using expectation maximization [67]. All LD plots as well as LD measurements and haplotype frequency calculations involved use of SVS v8.8.1. This collection consisted 220 PIs from Africa, 352 from Asia, 497 from Europe, 121 from North America, and 16 from South America [26].

### 4.5. Total RNA Extraction and qRT-PCR

Total RNA was extracted from watermelon flesh (three biological replicates) by using TRIzol^TM^ Reagent (Life Technologies, Carlsbad, CA, USA). On-column DNase I treatment was used to remove genomic DNA. The RNA concentrations and quality were determined by using NanoDrop 2000 (Thermofisher, Waltham, MA, USA). The first strand cDNAs were synthesized with 1 µg total RNA in a 20 µL reaction mixture with SuperScript^TM^ II Reverse Transcriptase (Thermo Fisher Scientific, USA) per the manufacturer’s instructions. cDNAs were then diluted by adding 180 µL sterile nuclease-free water. qRT-PCR was performed with PowerUp^TM^ SYBR Green Master Mix (Applied Biosystems, Thermo Fisher Scientific, USA) and the StepOnePlus^TM^ Real-Time PCR System (Applied Biosystems). PCR was programmed at 95 °C for 30 s, followed by 95 °C for 10 s and 60 °C for 60 s for 40 cycles. Gene expression was normalized to *Actin 8* expression (housekeeping reference), and transcript abundance of target genes was calculated with the PI 526238 accession, with significantly low citrulline content, as a calibrator. The 2^−ΔΔCT^ method was used to estimate relative transcript level [68]. The primers for qRT-PCR analysis are in Table S3.

## 5. Conclusions

This study demonstrated a wide variation in watermelon germplasm for citrulline content. Identifying such variability and introgressing it into high-yield lines will be key to long-term watermelon breeding for improved nutraceutical content. We found moderate to high (83%) broad-sense heritability, which indicates the possibility of successful citrulline enhancement with introgression breeding. Here we provide new insights into the domestication and population genetic structure based on citrulline content in geographically spread-out watermelon accessions. The SNP loci associated with citrulline content identified in this study would benchmark the efforts of development of molecular markers to enhance nutritional quality in elite watermelon cultivars.

## Figures and Tables

**Table 1:** The significant non-synonymous SNPs associated with citrulline content in watermelon flesh.

Marker	Locus1	*p*-Value	−log10	Regression Beta	Beta Standard Error	FDR	Minor Allele Frequency
S02_33508197	ClCG02G018770	0.00	3.18	6.95	1.99	0.10	0.17
S02_33508131	ClCG02G018770	0.00	3.34	6.42	1.78	0.10	0.19
S02_28460679	ClCG02G014160	0.00	3.33	6.57	1.82	0.09	0.19
S04_19161720	ClCG04G005470	0.00	4.29	6.79	1.61	0.09	0.25
S04_10803195	ClCG04G002830/ClCG04G002840	0.00	3.06	6.11	1.79	0.12	0.21
S04_19161725	ClCG04G005470	0.00	4.29	6.79	1.61	0.05	0.25
S06_30930976	ClCG06G017840	0.00	4.21	7.28	1.75	0.04	0.21
S06_30991451	ClCG06G017910	0.00	3.36	6.17	1.71	0.15	0.21
S07_12838412	ClCG07G006720	0.00	3.44	−4.70	1.28	0.16	0.49
S07_6258382	ClCG07G004850	0.00	3.25	−4.85	1.37	0.10	0.42
S09_9172194	ClCG09G009500	0.00	3.34	6.28	1.74	0.14	0.21
S10_19726131	ClCG10G008990	0.00	3.24	5.72	1.62	0.09	0.28

Abbreviations: FDR- false discovery rate.

**Table 2:** Gene ontology classification of the genes associated with significant SNPs.

Marker	Locus and SNP Location	Gene Annotation	Molecular Function	Biological Process	Cellular Component	Ma/Mi	Amino acid change
S02_33508197	ClCG02G018770-Intron	Ferrochelatase	Ferrochelatase activity	Heme biosynthesis	Cytoplasm	A/G	-
S02_33508131	ClCG02G018770-Intron	Ferrochelatase	Ferrochelatase activity	Heme biosynthesis	Cytoplasm	G/A	-
S02_28460679	ClCG02G014160-Exon	F-box/LRR-repeat protein 2	Protein binding	Protein destabilization	Nucleus	T/C	G→G
S04_19161720	ClCG04G005470-3′UTR	Golgi SNAP receptor complex 2	SNAP receptor activity	Transport	Golgi apparatus	C/T	F→F
S04_10803195	ClCG04G002830/ ClCG04G002840 Intergenic	DNA polymerase I	DNA binding	Regulation of transcription	Nucleus	C/A	-
S04_19161725	ClCG04G005470-3′UTR	Golgi SNAP receptor complex 2	SNAP receptor activity	Transport	Golgi apparatus	T/G	R→M
S06_30930976	ClCG06G017840-Intron	SAP domain-containing protein	DNA binding	Regulation of translation	Nucleus	C/A	
S06_30991451	ClCG06G017910-Exon	Acetolactate synthase	Valine biosynthesis	BCAA biosynthesis	Chloroplast	T/C	N→S
S07_12838412	ClCG07G006720-Exon	BAG family molecular chaperone regulator 1	Protein binding	Defense response to fungus,	Plasmodesma	A/C	L→W
S07_6258382	ClCG07G004850-Intron	TLC ATP/ADP transporter	ATP:ADP antiporter activity	Transport	Membrane	C/A	-
S09_9172194	ClCG09G009500-Intron	Protein of unknown function	–	–	–	T/A	-
S10_19726131	ClCG10G008990-Exon	Phototropic-responsive NPH3 family protein	Protein binding	Protein ubiquitination	–	G/C	W→C

Abbreviations: LLR- leucine-rich repeat; SAP- after SAF-A/B, Acinus and PIAS; BAG- B-cell lymphoma 2 associated athanogene 1; TLC- thin layer chromatography; NPH3- nonphototropic hypocotyl 3; SNAP- soluble NSF (N-ethylmaleimide-sensitive fusion protein) attachment protein; Ma/Mi- Major/Minor allele. The arrow (→) indicates amino acid change. 2.4. Functional Validation of Associated Genes.

## Supplementary Materials

Supplementary materials can be found at https://www.mdpi.com/1422-0067/20/21/5392/s1.

## Abbreviations

ALS	Acetolactate synthase
ASsu1	Acetolactate synthase small subunit_1
ASsu1	Acetolactate synthase small subunit_2
BCAAs	branched-chain amino acid synthesis
FDR	false discovery rate
FC	Ferrochelatase
GWAS	genome-wide association studies
IBD	identity by descent
LD	linkage disequilibrium
NJ	neighbor-Joining
PI	Plant Introduction
SNP	single nucleotide polymorphism
