# Supplementary material for: Haplotype Networking of GWAS Hits for Citrulline Variation Associated with the Domestication of Watermelon
Source: Int J Mol Sci. 2019 Oct 29;20(21):5392. doi: 10.3390/ijms20215392 (PMC6862219; doi:10.3390/ijms20215392)
Supplement: Supplementary file 1 [file ijms-20-05392-s001.zip › Supplementary Figure 1.pdf]

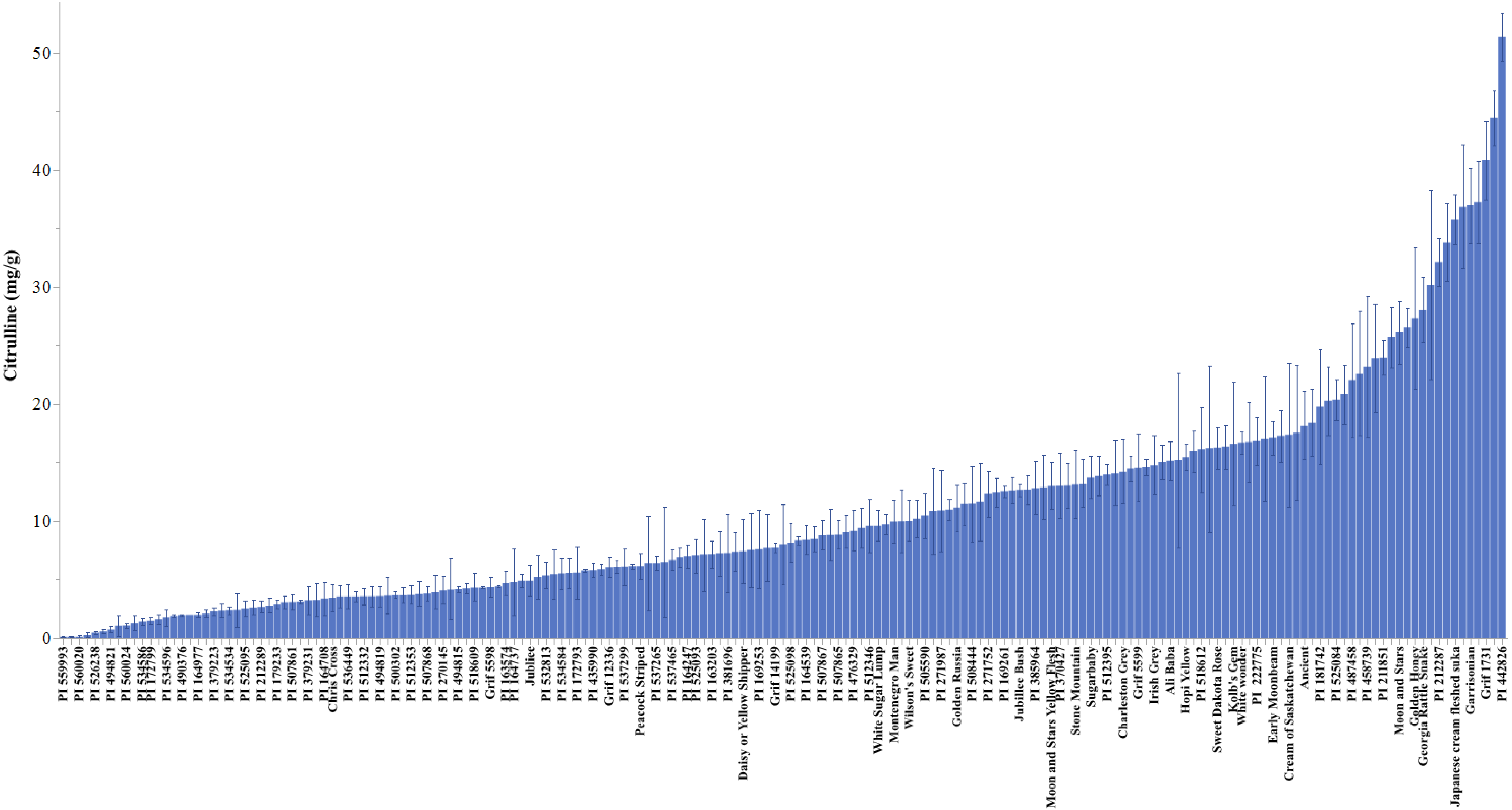

Supplementary Figure 1. Quantitative variation of citrulline content across watermelon accessions used in this study.
